# Autologous Fat Grafting in the Treatment of Painful Postsurgical Scar of the Oral Mucosa

**DOI:** 10.1155/2015/842854

**Published:** 2015-05-12

**Authors:** Andrea Lisa, Valeria Summo, Valeria Bandi, Luca Maione, Matteo Murolo, Francesco Klinger, Marco Klinger

**Affiliations:** ^1^Department of Medical Biotechnology and Translational Medicine BIOMETRA, Plastic Surgery Unit, Humanitas Research Hospital, Reconstructive and Aesthetic Plastic Surgery School, University of Milan, Rozzano, 20089 Milan, Italy; ^2^Plastic Surgery Unit, MultiMedica Holding S.p.A., Reconstructive and Aesthetic Plastic Surgery School, University of Milan, Sesto San Giovanni, 20099 Milan, Italy

## Abstract

*Background*. Persistent pain as a consequence of surgical treatment has been reported for several common surgical procedures and represents a clinical problem of great magnitude. *Material and Methods*. We describe the case of a 47-year-old female who presented a retractile scar that adhered to deep planes at the upper right of the vestibule due to surgical removal of maxillary exostosis, which determined important pain symptoms extending till the right shoulder during both chewing and rest. We subsequently treated her with autologous fat grafting according to Coleman's technique. *Results*. Clinical assessments were performed at 5 and 14 days, 1, 3, and 6 months, and 1 year after surgical procedure. We observed a progressive release of scar retraction together with an important improvement of pain symptoms. *Conclusion*. The case described widens the possible application of autologous fat grafting on a new anatomical site as buccal vestibule and in one specific clinical setting confirming its promising biological effects.

## 1. Introduction

Exostoses are nodular protuberances of mature bone whose precise designation depends on anatomic location.

Buccal exostoses occur along the buccal aspect of the maxilla or mandible, usually in the premolar and molar areas. Palatal exostoses are found on the palatal aspect of the maxilla, and the most common location is the tuberosity area. These entities could become symptomatic when they reach such a volume to interfere with feeding and speaking or to alter facial mimic and contour.

The histologic features of exostoses are described as hyperplastic bone, consisting of mature cortical and trabecular bone [1]. Their etiology is still under debate.

When being symptomatic, exostosis allows the possibility of surgical removal that could be related to different drawbacks.

Postsurgical scar retraction should be mentioned together with pain sensation during chewing, which can compromise dramatically the quality of life of patients.

Up to now no support therapy has been described to treat these weakening symptoms in this group of patients.

Autologous fat grafting is a relatively new technique which has been recently adopted to treat various pathologic conditions in reconstructive surgery, such as scars and burn keloid [2–7], and pain syndromes such as postmastectomy pain syndrome (PMPS) [8, 9] and in the treatment of Arnold neuralgia [10].

Moving from these evidences, we decided to adopt autologous fat grafting for the treatment of postsurgical scar retraction and pain sensation related to exostoses surgical removal, in order to verify its possible beneficial effects in this new approach.

## 2. Case Presentation

A female patient, aged 47 years, came to Department of Plastic Surgery at Humanitas Research Hospital in February 2013. She was referred to the department for surgical removal of maxillary exostosis in 1999 and related to the surgery a scar adhered to deep planes at the upper right of the vestibule which determined important pain symptoms extending till the right shoulder both during chewing and at rest, interfering with feeding and speaking.

Moreover the patient revealed chronic assumption of analgesic medication (Ibuprofen 600 mg) to control pain sensation. She did not assume any other medications.

Clinical history revealed a diagnosis of Sjogren syndrome and corrective surgery of cleft palate in 1996; no other pathologic conditions were present.

Clinical examination showed a postsurgical scar area of about 2 cm in length, retracted and adherent to deeper planes just at the upper right of the vestibule, which causes pain at digital pressure.

After collection of both clinical history and examination we proposed to our patient surgical scar tissue correction with autologous fat grafting.

**Figure 1:**
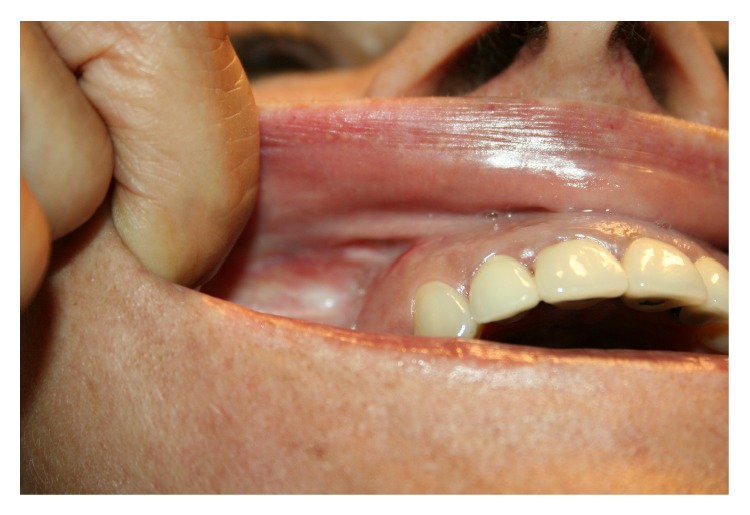
Preoperative image.

Our patient was informed about surgical procedure in particular regarding fat grafting unpredictable reabsorption rate and clinical results in this particular case. Both informed consent form and preoperative images were collected (Figure 1).

After routine preoperative examination and clinical assessment, the patient underwent liposuction under sedation and local anesthesia. The adipose tissue was harvested from the right flank, which is an easy accessible and abundant reservoir of adipose tissue.

**Figure 2:**
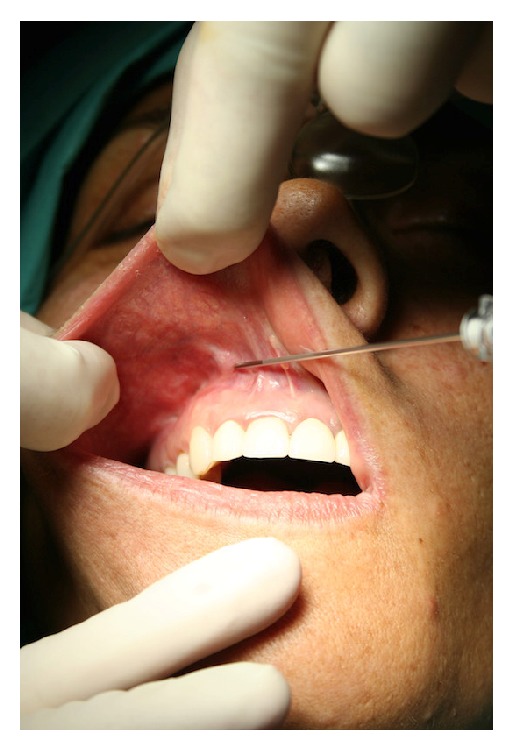
Intraoperative picture.

Following Coleman's procedure [11], the obtained fat was processed by centrifugation at 3000 rpm for 3 minutes. The fat graft was injected using an 18-gauge angiographic needle with a snap-on wing (Cordis, a Johnson & Johnson Company, NV, Roden, Netherlands) under mucous membrane in the scar area at the upper right vestibule (Figure 2). A total of about 5 cc of adipose tissue was injected.

Following surgery pressure dressing was applied over donor site for 5 days and antibiotic therapy was recommended for 5 days (cefixime 400 mg 1 pill per day).

Clinical assessment was performed after surgical procedure at 5 and 14 days, 1, 3, and 6 months, and 1 year.

**Figure 3:**
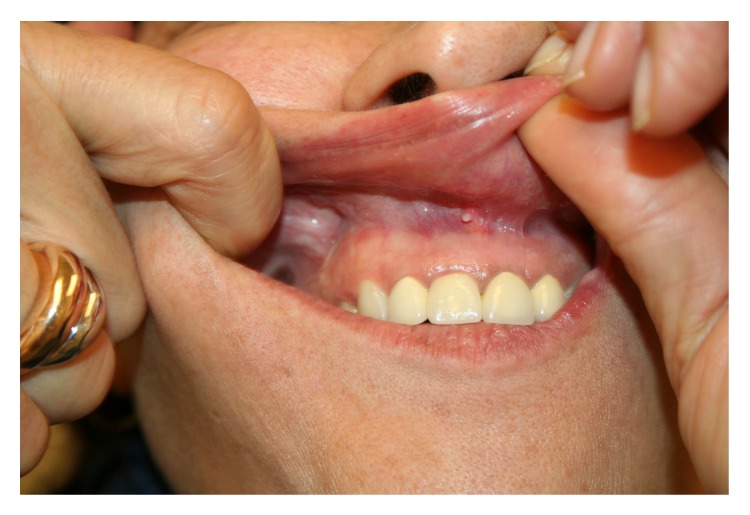
Postoperative image.

During the clinical meeting we observed progressive release of scar retraction and quality improvement measured with POSAS scale [12], together with an important decrease of pain symptoms which lasts for all the postoperative follow-up controls (Figure 3).

**Figure 4:**
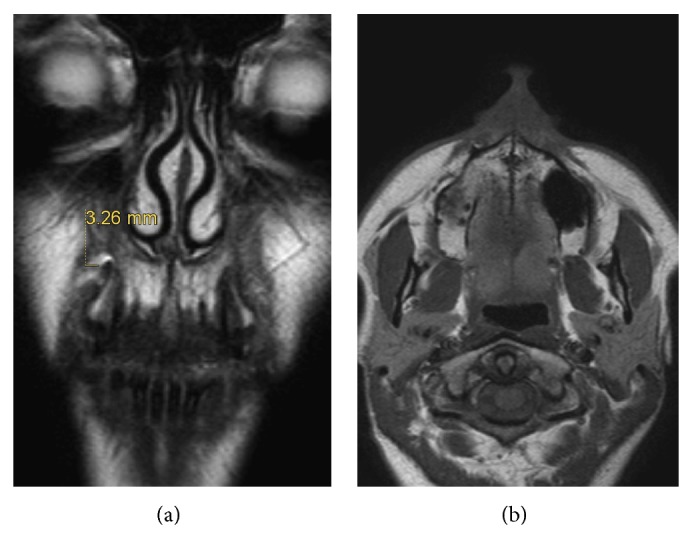
(a)-(b) MRI of the facial skeleton after 3 months from surgery. We can appreciate soft tissue volume deposition in the area of previous fat grafting.

After 3 months from surgical operation we performed an MRI of the facial skeleton and we appreciated a soft tissue volume increase in the area of previous fat grafting (Figure 4). No local or systemic signs of infection were found, and no complications occurred. Moreover the patient declared that she stopped analgesic drug assumption immediately after operation.

## 3. Discussion

Exostoses have been described as nodular protuberances of mature intraoral bone.

Palatal exostoses revealed a prevalence of 30% while buccal exostoses presented a prevalence rate of 0.9 per 1000 persons [13].

These clinical entities could become invalidating for patients especially when they reach such a volume to interfere with feeding and speaking or to alter facial mimic and contour.

In these advanced cases surgical treatment is needed to remove bony prominences and to restore oral function and contour. Nonetheless also surgical removal could be related to different drawbacks. In fact postsurgical scar retraction could determine chronic pain in oral region, in particular during chewing, with an overall reduction of patients' quality of life.

No support therapy has been described until now for these weakening symptoms in this group of patients who, most of the time, are analgesic medications dependent.

Persistent pain as a consequence of surgical treatment has been reported for several common surgical procedures and represents a clinical problem of great magnitude.

Our team adopts autologous fat grafting in the treatment of multiple pathological status beyond all scar treatment and pain syndromes.

We start treating postmastectomy pain syndrome obtaining significant decreases of neuropathic pain [8, 9].

Supported by our promising results we approached with positive results also chronic headaches of cervical origin, both chronic cervicogenic and occipital neuralgia [10].

Moving from these evidences we consider autologous fat grafting as an innovative solution for pain syndromes related to scar retraction although the exact mechanism of action is still unclear.

Laboratory findings have demonstrated the presence of mesenchymal multipotent stem cells in the adipocyte cell fraction of fat graft [14].

We hypothesize that autologous fat graft due to the regenerative role of the stromal fraction of adipose tissue grafted could promote a reorganization of fibrotic tissue together with soft tissue regeneration, leading to scar release and reducing nerve excitatory pattern with consequent positive clinical results on pain control.

In addition to that, surgery-associated tissue injury leads to an inflammatory reaction accompanied by increased production of proinflammatory cytokines, which can induce peripheral and central sensitization with a failed nociception system, leading to pain augmentation.

Mesenchymal stem cells and adipose-derived stem cells could efficiently reduce T-cell activation inhibiting the proliferation of CD4 and CD8 T lymphocytes [15]. It could be hypothesized that fat grafting could inhibit inflammation and determine pain reduction and analgesia.

The case reported showed a positive final outcome. Buccal vestibule scar retraction showed a release after one procedure of autologous fat grafting and our patient referred to clinical remission of pain which did not need any analgesic treatment with a total follow-up of one year.

The case described widens the possible application of autologous fat grafting on a new anatomical site as buccal vestibule and in one specific clinical setting confirming its promising biological effects.

In fact our experience could open a new route of regenerative surgery addressing a mucosal tissue as buccal one.
